# Design of a Tri-Axial Surface Micromachined MEMS Vibrating Gyroscope

**DOI:** 10.3390/s20102822

**Published:** 2020-05-15

**Authors:** Rocco Crescenzi, Giuseppe Vincenzo Castellito, Simone Quaranta, Marco Balucani

**Affiliations:** 1 Department of Information, Electronic and Telecomunication Engineer, Sapienza University of Roma, 00184 Rome, Italy; simone.quaranta@uniroma1.it (S.Q.); marco.balucani@uniroma1.it (M.B.); 2 Department of Mechanical and Aerospace Engineer, Sapienza University of Roma, 00184 Rome, Italy; castellitto.1485567@studenti.uniroma1.it

**Keywords:** vibrating gyroscope, MEMS, comb-drive, micromachining, cantilever, compliant elements

## Abstract

Gyroscopes are one of the next killer applications for the MEMS (Micro-Electro-Mechanical-Systems) sensors industry. Many mature applications have already been developed and produced in limited volumes for the automotive, consumer, industrial, medical, and military markets. Plenty of high-volume applications, over 100 million per year, have been calling for low-cost gyroscopes. Bulk silicon is a promising candidate for low-cost gyroscopes due to its large scale availability and maturity of its manufacturing industry. Nevertheless, it is not suitable for a real monolithic IC integration and requires a dedicated packaging. New designs are supposed to eliminate the need for magnets and metal case package, and allow for a real monolithic MEMS-IC (Integrated Circuit) electronic system. In addition, a drastic cost reduction could be achieved by utilizing off-the-shelf plastic packaging with lead frames for the final assembly. The present paper puts forward the design of a novel tri-axial gyroscope based on rotating comb-drives acting as both capacitive sensors and actuators. The comb-drives are comprised of a single monolithic moving component (rotor) and fixed parts (stators). The former is made out of different concentrated masses connected by curved silicon beams in order to decouple the motion signals. The sensor was devised to be fabricated through the PolyMUMPs^®^ process and it is intended for working in air in order to semplify the MEMS-IC monolithic integration.

## 1. Introduction

Gyroscopes are angular velocity sensors employed in several industrial applications such as inertial navigation, automotive control, rollover detection, etc. [1]. The first gyroscopes would rely on rotating wheels in order to measure the angular velocity. Current gyroscope technology is mainly predicated on optical methods. Indeed, angular velocity is measured through the phase difference between two optical sources traveling in opposite directions. Despite their large size and price, optical-based gyroscopes are attractive because of the high precision provided in terms of rotational rate information, robustness, and reliability.

The miniaturization of sensors and actuators represents a major challenge since the 1990s. Needless to say, size reduction from macroscopic to microscopic scale brings about changes in the mechanical properties of materials and impacts on the mechanical behavior of the final device [2,3,4].

MEMS gyroscopes (MEMS-Gs) are characterized by small dimensions, lightweight structures, and low production costs. These features make MEMS-Gs ideal for plenty of applications from consumer electronics to high-tech devices. Furthermore, the lack of rotating parts renders MEMS gyroscopes suitable for large scale miniaturization and mass production through conventional IC manufacturing technologies (e.g., batch micromachining). Usually, MEMS-Gs are grouped in three categories, namely *Vibratory gyroscopes* [5,6], *Tuning fork gyroscopes* [7,8], and *Vibrating-Wheel gyroscopes* [9,10]. Most of them are based on the energy transfer between two orthogonal vibration modes: the drive-mode and the sense-mode. The drive-mode constitutes the electrostatically-actuated component of the device and the sense-mode is activated only when a Coriolis Force arises. Thus, the amplitude of the sense mode is proportional to the measured angular velocity [11].

Flexural elements are often exploited in MEMS application to attain large structural mobilities and displacements [12]. For instance, the authors of [13,14,15] have used flexural elements (e.g., U-beams) for both drive mode vibration and sense mode activation. On the other hand, the Verotti et al. [16], Balucani et al. [17], and Belfiore et al. [18] have reported the kinematic and dynamic behavior of a microgripper relying on movable parts constrained by hinges based on high-mobility curved flexural elements. Finally, long flexural beams anchored at both edges have also been used to generate in-plane and out-of-plane mass displacements [19].

Pull-ins in curved comb-drives are generally harder to avoid than in linear ones. In fact, detailed kinematic and dynamic studies are required in order to understand the effects of the structure on the electrostatic field. For example, Crescenzi et al. have shown that [20] the geometry of comb-drives fingers is of paramount importance to prevent the pull-in effect and achieve wide angular rotations. These findings have also been analyzed by Yeh et al. [21] and Chang et al. [22], who also explored the relationship between pull-in and system geometry. The former has mostly focused mostly on the mechanical stability of fingers, whereas the latter has accomplished the electrostatic calculation of the capacitive force and torque generated by the comb-drive upon the application of a voltage.

This work proposes the design of a MEMS gyroscope amenable to surface micromachining manufacturing and MEMS-IC monolithic integration. The design aims to combine electromechanical performances comparable to bulk silicon technology with low production costs. The gyroscope is composed of a monolithic “rotating” part (rotor) and multiple fixed parts (stators). Moreover, it employs comb-drives for both the actuated and the sensing components. The rotor is made out of different concentrated masses connected by curved silicon beams allowing for signal decoupling. Flexural elements and comb-drives comprising curved fingers are instrumental in implementing rotational motion. The sensor is meant to be fabricated by means of the PolyMUMPs^®^ process and it is intended for operation in air. The whole design is devoted to simplifying the MEMS-IC monolithic integration.

## 2. Gyroscope Fundamentals

**Figure 1:**
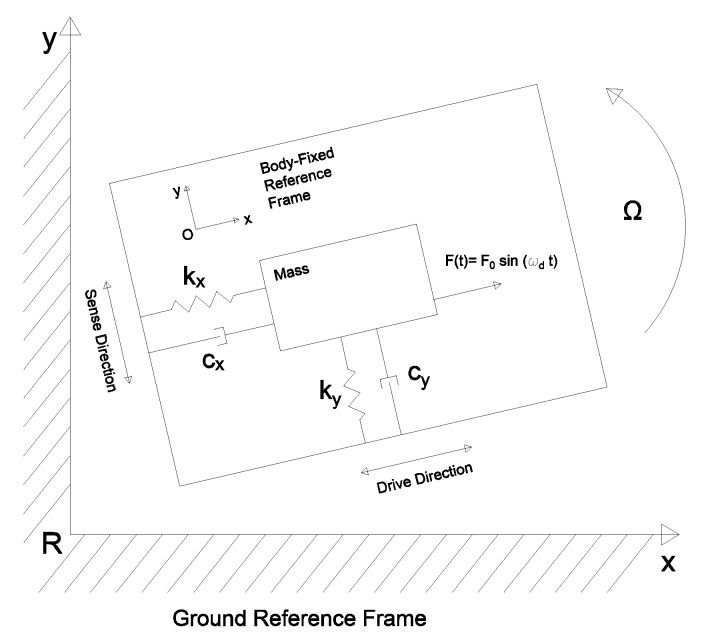
Gyroscope schematic. Sense and drive mode are illustrated.

The overall structure of a gyroscope (see Figure 1) is comprised of two distinct but interrelated parts [23,24,25]:

- The drive-masses ($m_{d}$) providing structural support to the sensor. Drive-masses account for the main contribution to the resonance frequency. Drive-masses excitation occurs by an electrostatic actuation. Drive-masses displacement ($x_{d}$) is called drive-mode.
- The sense-masses ($m_{s}$) detecting the Coriolis force. Indeed, the displacement of the sense-masses ($x_{s}$) is called sense-mode and it is proportional to the magnitude of the force acting on them.

The drive-mode may affect the sense-mode resulting in the “quadrature” error that is already taken into account in the design of the present sensor.

As long as no directional coupling between the drive- and the sense-modes occurs, the motion of the sensor (at a frequency ω) can be described by the equations below. For the drive mode:

(1) $$\Theta_{d}=H_{d}\left(\omega \right)T_{d}$$

(2) $$H_{d}\left(\omega \right)=\frac{1}{-\omega^{2}+j\omega \omega_{d}\zeta +\omega_{d}}$$

where $\Theta_{d}$ is the rotation angle, $T_{d}$ is the torque applied to the structure and, for the transfer function $H_{d}\left(\omega \right)$, $\omega_{d}$ is the resonant frequency, and ζ is the damping ratio. For the sense mode:

(3) $$x_{s}\left(\omega \right)=2\omega m_{d}\Omega H_{s}\left(\omega \right)$$

where Ω is the angular velocity of the frame, $m_{d}$ represents the mass of the drive elements and $H_{s}$ is the transfer function of the sense movement.

Two different sense-mode cases exist, depending on the oscillation frequency of the sense masses ($\omega_{s}$). When the natural oscillation frequencies of the two modes match (i.e., matching mode operation, $\omega_{d}=\omega_{s}$) the sense-mode response is amplified by the sense-mode quality factor $Q_{s}$ (Equation (4)):

(4) $$x_{s}=-2\frac{m_{d}}{m_{s}}\frac{Q_{s}}{\omega_{s}}\Omega x_{d}$$

where $m_{s}$ represents the mass of the sense elements. Although huge signal gains are possible, matching mode operation leads to narrow bandwidths and requires electrical or physical trimming. Conversely, when the sense-mode resonant frequency is considerably larger than the drive-mode one (i.e., separate modes, $\omega_{d}<<\omega_{s}$) the displacement is given by (Equation (5)):

(5) $$x_{s}=-j\frac{2\omega m_{d}x_{d}}{k_{s}}\Omega$$

where $k_{s}$ is the elastic constant for the sense mode the stiffness of the sensing elements. Usually the phase difference between the two modes is smaller than $90^{\circ }$. Hence, the detected Coriolis force can be easily decoupled from the drive-modes (i.e., the quadrature error tends to vanish). Furthermore, large mechanical bandwidths compared to the matching mode case can be attained.

## 3. System Description

**Figure 2:**
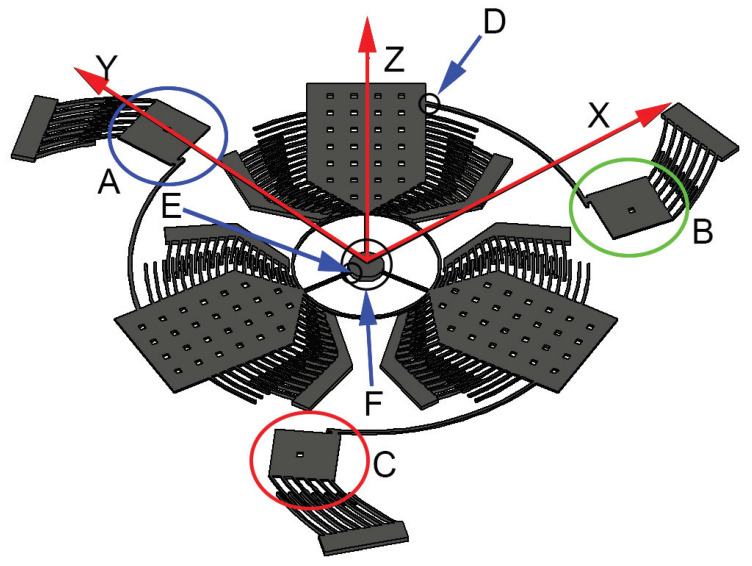
Pictorial representation of the tri-axial gyroscope design.

The gyroscope presented in this paper (see Figure 2), is comprised of three drive-masses positioned $120^{\circ }$ apart from each other. The $120^{\circ }$ separation minimizes the number of driving masses while keeping a symmetric structure. Furthermore, the $120^{\circ }$ condition allows the ability to cancel the overall Coriolis force acting on the driving masses when an angular velocity along the z-axis is considered. No other axes orientation allows for such condition with only three drive-masses. The three drive-masses are connected to the same annular support to guarantee the same displacement.

The stiffness and robustness of the structure are provided by three cantilever beams jointed to the silicon substrate through a torsional beam (the pivotal point and the substrate are indicated as E and F in Figure 2, respectively). The three squared sense-masses (indicated as A, B, C in Figure 2) were placed at each free end of the drive masses in order to maximize their distance from the center of rotation, their velocity, and ultimately the resulting Coriolis force. Besides, sense-masses velocity and the resulting Coriolis force are also maximized. Three cantilever beams connect the sense masses to the drive-masses (the joint is indicated as D in Figure 2). The stiffness of the cantilever allows the system to operate in matched modes when the masses detect an angular velocity along the *z*-axis and in separated modes when an angular velocity is sensed along the *y* and *x* axes. The displacement is measured by means of capacitive sensing. Two kinds of sense-mass displacement can be detected depending on the direction of the angular velocity:

- When the angular velocity is oriented along the *z*-axis a rotation about the joint between the sense and the drive mass arises. Since the measurement is carried out through comb-fingers, matched modes are mandatory to obtain a significant signal boost.
- A vertical displacement appears for angular velocities along the *y* or the *x* axes. The mass exhibiting the maximum displacement among the three reveals the direction of the rotation. In addition, the direction of the angular velocity can be inferred from the upward or downward displacement of the other two masses. Separated modes are exploited to avoid collision between the masses and the silicon substrate.

The actuation is performed by using rotary comb-drives. An actractive force between the stator and the rotor of the sensor is created upon the application of a voltage between the two elements. Consequently, a torque acting on the structure is generated. An oscillating voltage maintaining a π phase difference between the left and right comb-drives is applied to sustain the vibration of the structure.

The characteristics of the model device used for numerical simulations are summarized in Table 1 and Table 2:

## 4. Numerical Modeling of the Structure

The design of the sensor was accomplished through a Matlab-lumped algorithm. Simulations were conducted neglecting some aspects that can stem from silicon anisotropy [26]. From a first-guess solution, the algorithm calculated the stiffness and the mass matrices by splitting the sensor into its fundamental components:

- drive-masses
- cantilever-springs
- sense-masses
- torsional spring

Then, the resonance frequency was derived from the stiffness and the mass matrices by solving Equation (6):

(6) det([K]−λ×[M])=0

Finally, sense-masses displacement, sensitivity curves, and the minimum torque required to keep the sensor rotation going were calculated by modal-analysis. The algorithm was set to change the dimensions of the system over and over until the output parameters would comply with all the initial constraints.

### 4.1. First Guess Solution

A first guess solution predicated on the following assumptions was found in order to cut down on computational time:

- The link between the sense-masses and the drive masses is perfectly rigid. The same condition holds for the silicon substrate-gyroscope connection.
- The moment of inertia of smaller parts is immaterial.
- The bores of the drive and sense masses are not taken into account.

#### 4.1.1. First Design of the Drive Mass

Drive mode is related to the other salient parameters of the system (the Coriolis force, the drive and sense forces, the inertia forces, and the damping) by the following equation (Equation (7)):

(7) $$I\ddot{\theta }\;+b_{\theta }\;\dot{\theta }\;\dot{+}k\theta =T_{c}+T_{d}+T_{s}$$

where I is the moment of inertia, $b_{\theta }$ is the damping, k is the stiffness, $T_{c}$ is the torque resulting from the Coriolis force, $T_{d}$ is the torque caused by the drive actuation, and $T_{s}$ is the torque stemming from sensing. After Fourier transform, the previous equation becomes (Equation (8)):

(8) $$-I\omega^{2}\Theta +2j\omega \zeta \Theta +k\Theta =T_{c}\left(\omega \right)+T_{d}\left(\omega \right)+T_{s}\left(\omega \right)$$

The first natural frequency can be computed as usual (Equation (9)):

(9) $$\omega_{n}=\sqrt{\frac{k}{I}}$$

Thus, the frequency response function is (Equation (10)) [27]:

(10) $$G\left(\omega \right)=\frac{1}{\left(1-\frac{\omega^{2}}{\omega_{n}^{2}}+j\frac{2\zeta \omega }{\omega_{n}}\right)}$$

and the frequency response function modulus is (Equation (11)):

(11) $$\left|G\left(\omega \right)\right|=\frac{1}{\sqrt{{\left(1-\frac{\omega^{2}}{\omega_{n}^{2}}\right)}^{2}+{\left(\frac{2\zeta \omega }{\omega_{n}}\right)}^{2}}}$$

which shows a maximum for $\omega =\omega_{n}$. Two different beam springs ensure the stiffness of the structure. A cantilever beam connects the drive-mass to a pivot that, in turn, provides the structural connection to the silicon substrate. The overall stiffness was calculated by modeling the whole structure as a system of parallel springs. The stiffness of both the annulus and the drive-masses were neglected. Indeed, both components are usually stiffer than the cantilever and can be considered rigid bodies. The stiffness of a set of parallel-connected springs is described by (Equation (12)):

(12) $$\frac{1}{K_{eq}}=\frac{1}{K_{1}}+\frac{1}{K_{2}}+\cdots +\frac{1}{K_{n}}$$

#### 4.1.2. First Design of the Sense Masses

Despite the simple shape of the sense masses, their design it is not trivial. Indeed, they should be small enough to provoke a substantial change in the resonance frequency of the structure. Nonetheless, the masses should still be large enough to allow for displacement sensing and to hold the sensing-comb drive. Sense-masses were chosen to be squared and linked to curved beams for achieving high velocities. The length of the sense masses was set to half the drive-masses value, whereas the cantilever design was adjusted to resonate at the same frequency of the drive masses. The resonant frequency of the sense masses was, in turn, matched to the one of the drive masses in the radial modes.

### 4.2. Iterative Design

Once a first guess solution had been found, it could be inserted into the algorithm to calculate the displacement of the sense-masses and the change in the capacitance with respect to angular velocity variations. The inputs in the algorithm were the resonance frequency, the PolyMUMPs^®^ process constraints, and the first guess solution.

The standard PolyMUMPs^®^ process resolution is 1 μm. Thus, each and every dimension in the design had to be rounded to the nearest natural number. This restriction called for an iterative procedure. The iteration was carried out by an algorithm developed in MATLAB. Besides the fabrication process specifications, several other requirements were to be met to guarantee the functioning of the sensor in order for the sensor to work properly:

- The out of plane displacement should not exceed 1 μm.
- The capacitance variation between the sense masses and the ground should be over 1 aF in order to make the measurement feasible.
- A safety factor greater than five should be set to prove the resistance of the structure. The safety factor is defined as the ratio between the calculated stress of the structure and the maximum bearable stress of the material.

Furthermore, improvements to the algorithm were needed to overcome some of the initial approximations used during the first guess solution stage:

- The inertia of the sense masses and their contribution to the resonant were also considered in the resonant frequency formula.
- The inertia of the triangular connector was also taken into account.

The algorithm was fed with both PolyMUMPs^®^ and first guess solution conditions to calculate the resonance frequency and verifying the matching of all the constraints. When the aforementioned requirements were not met or the resonant frequency was out of the tolerance range, the algorithm would “tune” one parameter at the time until fulfilling all the conditions. The calculation also assumed a rotation of the drive-masses equal to $1^{\circ }$. The smallest value was picked out among a set of solutions satisfying the required constraints. The model computed the eigenfrequency as det(K−MI)=0, where K is the stiffness matrix, M the mass matrix, and I the identity matrix. Finally, a dumping model was introduced to calculate the displacement under resonant conditions.

#### 4.2.1. Damping

Generally, two main type of damping are found in MEMS structures [28]:

- Slide film damping
- Squeeze film damping.

The former takes place when two parallels plates are in a relative lateral motion with each other, while the latter manifests when relative vertical motion occurs [29].

Couette flow comes about when a constant velocity gradient across the fluid gap (i.e., laminar flow) exists. In fact, tangentially moving surfaces can be described through a Couette flow (i.e., nearly constant velocity gradient), as long as the motion takes place in the low frequencies range. The damping coefficient is given by (Equation (13)):

(13) $$c_{Couvette}=\frac{\mu A}{d}$$

where μ is the viscosity of the fluid, *A* is the surface area, and *d* is the gap between the two plates. On the other hand, velocity gradient condition does not hold a constant velocity gradient cannot be maintained in the Stokes flow regime. The transition between Couette and Stokes flow takes place near the cut-off frequency $f_{c}$ (Equation (14)):

(14) $$f_{c}=\frac{\mu }{2\pi \rho d^{2}}$$

where ρ is the density of the fluid. The Stokes-type damping affecting the plate can be expressed by (Equation (15)):

(15) $$C_{stokes}=\mu \;A\eta \frac{\sinh\left(2\eta d\right)+\sin(2\eta d)}{\cosh\left(2\eta d\right)-\cos\left(2\eta d\right)}$$

where $\eta =\sqrt{\pi f/\nu }$ and ν is the kinematic viscosity.

The fluidodynamic behavior changes as gas rarefaction changes. Thus, an effective viscosity is introduced (Equation (16)):

(16) $$\mu_{eff}=\frac{\mu }{1+2K_{n}}$$

where $K_{n}$ is the Knudsen number and it is defined as the ratio between the mean free path of gas molecules (or atoms) and the gap height. μ is the viscosity at small Knudsen number ($K_{n}<<0.1$).

Squeeze film damping describes the gas behavior between two surfaces moving toward each other. As the gap between the plate and substrate is decreased, the gas begins flowing from underneath the plate. If the movement of the plate is slow, the gas squeezes out provoking a dissipation loss. Conversely, if the plate moves fast enough a spring force also acts on the plate. Moreover, the pressure of the plate also results in an increase of the mass impinging on the the plate. The effective viscosity from the squeeze film damping is (Equation (17)):

(17) $$\mu_{eff}=\frac{\mu }{1+9.638{K_{n}}^{1.159}}$$

the damping coefficient is expressed by (Equation (18)):

(18) $$\gamma =\frac{64\sigma pA}{\pi^{6}d\omega }\sum_{m,\;n\;odd}\frac{m^{2}+c^{2}n^{2}}{{\left(mn\right)}^{2}\left[{\left(m^{2}+c^{2}n^{2}\right)}^{2}+\sigma^{2}\right]}$$

in the previous (Equation (18)) σ is defined as:

(19) $$\sigma =\frac{12{\mu_{eff}}^{2}W^{2}}{pd^{2}}\omega$$

In Equations (18) and (19) *m* and *n* are odd integers, *p* is the pressure, *d* is the plates distance and *c* = *W*/*L* is the ratio between the width (*W*) and the length (*L*) of the plates. The stiffness is calculated by means of (Equation (20))

(20) $$k=\frac{64\sigma^{2}pA}{\pi^{8}d}\sum_{m,\;n\;odd}\frac{1}{{\left(mn\right)}^{2}\left[{\left(m^{2}+c^{2}n^{2}\right)}^{2}+\sigma^{2}/\pi^{4}\right]}$$

As shown in the previous equations, the squeeze film is very sensitive to the gap width (*d*) and to the plate area (*A*).

After calculating the displacement of the sense masses, the algorithm computes the actuation configuration in terms of voltage, number of comb-drives, and expected sensitivity curves.

#### 4.2.2. Actuation

The main type of force exploited in MEMS devices is the electrostatic attraction (or repulsion) [30]. Among the electrostatic transduction methods, capacitive transduction is widely employed because of the simplicity of the fabrication process involved and low power consumption; however, large voltages may be needed to achieve appreciable forces. The electrostatic force responsible for capacitive transduction can be calculated from the parallel plate capacitor model. Such a fundamental configuration can be then modified to suit different types of capacitive actuators.

**Figure 3:**
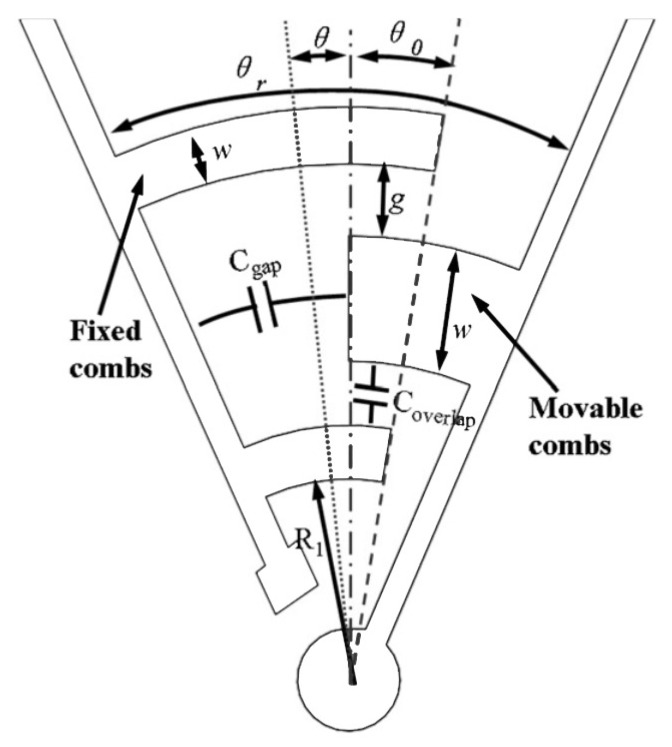
Example of a rotary comb-drive.

Comb-drive actuators rely on a voltage applied between the static and moving combs in order to draw them together. The resulting force is proportional to the the capacitance change across the two combs and increases as either the driving voltage or the number of comb teeth, or the gap between the teeth are increased. In-plane rotations are implemented in the driving-mode through rotary comb-drives (see Figure 3).

Rotary capacitors were regarded as parallel plates ones in our model. Besides, mass and material effects like inertia and dumping could be reasonably neglected. The overall capacitance arises from two sources: $C_{overlap}$ between the side wall surfaces and $C_{f}$ between the finger free-end sections and the inner stator surface. Other important geometrical parameters used in the model are:

- $R_{1}$, the inner radius of the closest finger with respect to the center of rotation.
- g, the finger gap.
- *W*, the finger width of the fixed comb fingers.
- t, the finger and curved beam thickness.
- $\theta_{0}$, the initial overlapping angle of the fingers.
- θ, the generated overlapping angle.
- n, number of fingers in a comb.
- $\theta_{r}$ initial stator-to-rotor opening sector angle.

$C_{overlap}$ and $C_{f}$, are given by Equation (21) and Equation (22), respectively.

(21) $$\begin{array}{cc} \; & A_{i}={\left[ln\frac{R_{1}+2\left(i-1\right)\left(W+g\right)}{R_{1}+2\left(i-1\right)\left(W+g\right)-g}\right]}^{-1}\\ \; & B_{i}={\left[ln\frac{R_{1}+\left(2i-1\right)\left(W+g\right)}{R_{1}+2\left(i-1\right)\left(W+g\right)+W}\right]}^{-1}\\ \; & C_{overlap}=\varepsilon_{0}t\left(\theta +\theta_{0}\right)\sum_{i=1}^{n-1}\left\{A_{i}+B_{i}\right\}-\varepsilon_{0}t\left(\theta +\theta_{0}\right){\left(ln\frac{R_{1}}{R_{1}-g}\right)}^{-1} \end{array}$$

(22) $$\begin{array}{cc} \; & D=\sum_{i=1}^{n}\left[\frac{1}{R_{1}+2\left(i-1\right)\left(W+g\right)}+\frac{1}{R_{1}+\left(2i-1\right)\left(W+g\right)}\right]\\ \; & C_{f}=\varepsilon_{0}tW\left(D-\frac{1}{R_{1}}\right)\frac{2-\theta }{\theta_{r}-\theta_{0}} \end{array}$$

**Figure 4:**
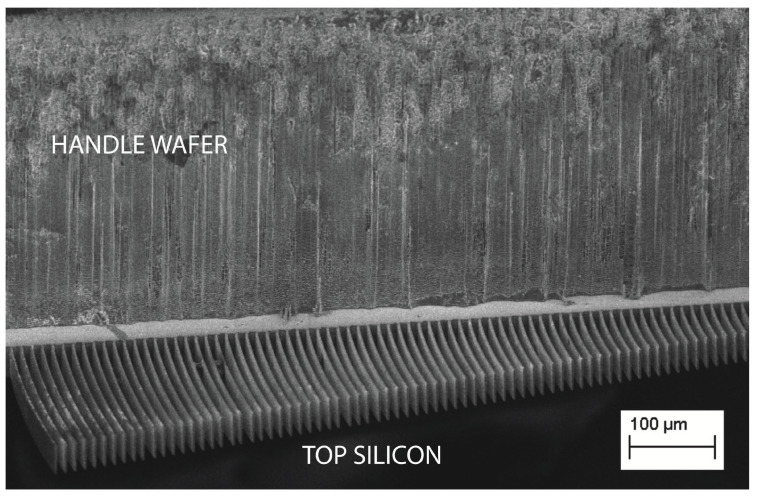
A SEM image showing the comb-drive fingers attached to an SOI (Silicon on Insulator) wafer.

**Figure 5:**
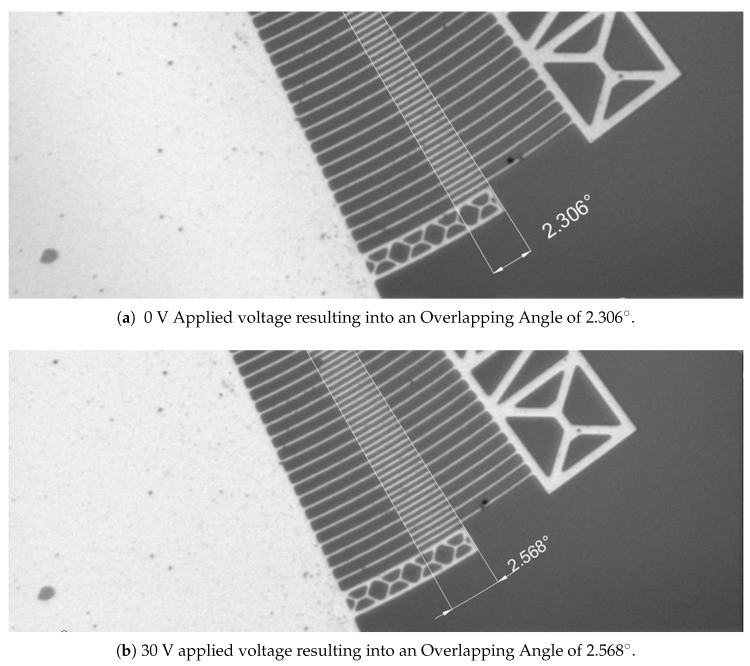
Comb-drive at starting and final positions.

**Figure 6:**
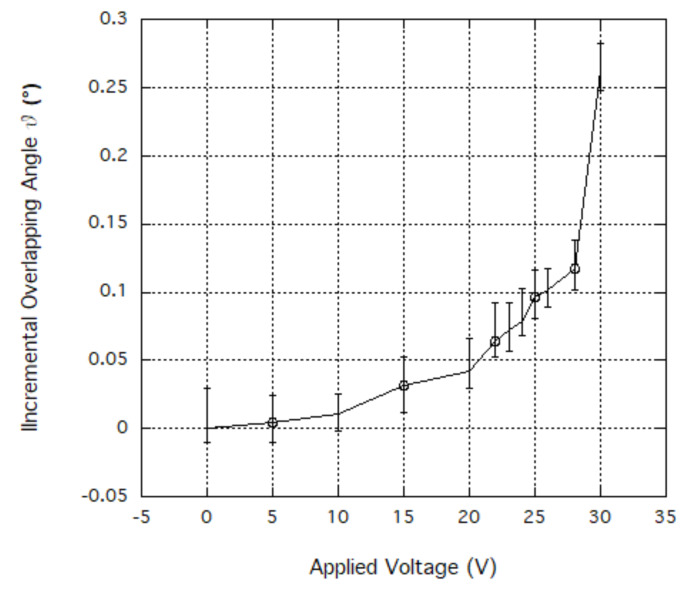
Behavior of Incremental Overlapping Angle vs Applied Voltage (V).

In order to validate the theoretical predictions, a comb-drives prototype (Figure 4) on a 475 μm thick SOI (Silicon on Insulator) wafer was fabricated by DRIE (Deep Reactive Ion Etching). The silicon layer was 40 μm thick (with 3 μm of buried oxide). The device was tested for actuation at 0 and 30 V. Figure 5 and Figure 6 show the behavior of the generated overlap angle as the applied voltage increases.

#### 4.2.3. Actuation Design

**Figure 7:**
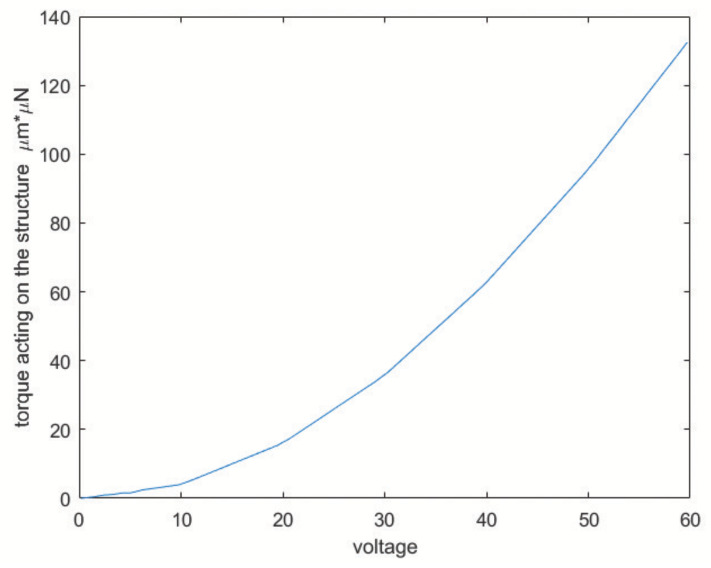
Torque versus voltage trend.

The torque needed to keep the vibration of the whole structure going is 74 μN ×μm. The force is applied on each of the three drive-masses; each set of comb-drives should provide a third of the total torque. According to the PolyMUMPs^®^ fabrication constraints each element needs to be spaced at least 2 μm from the next. Since the electrostatic force is inversely proportional to the distance between the comb-drive fingers, the minimum spacing between the elements of the structure was chosen. Fifteen comb-fingers and a $15^{\circ }$ opening angle between the drive-mass and comb-finger joint were used. Figure 7 shows the torque versus voltage graph. The desired torque value corresponds to a voltage of about 40 V.

When a voltage *V*${}_{c}$ is applied to the comb-drive, the electrostatic force tends to reduce the gap. For low voltages this effect is countered by the spring forces; however, when the voltage increases too much the fixed and moving part eventually snap together. Thus, an estimate of the maximum acceptable voltage (i.e., to avoid pull-in) is fundamental for the successful design of electrostatic actuators. The pull-in voltage *V*${}_{p}$ can be calculated as (Equation (24)):

(23) $$\begin{array}{c} V_{c}=\sqrt{\frac{2kd^{3}}{\varepsilon S}} \end{array}$$

(24) $$\begin{array}{c} V_{p}=\sqrt{\frac{4}{27}}V_{c} \end{array}$$

**Figure 8:**
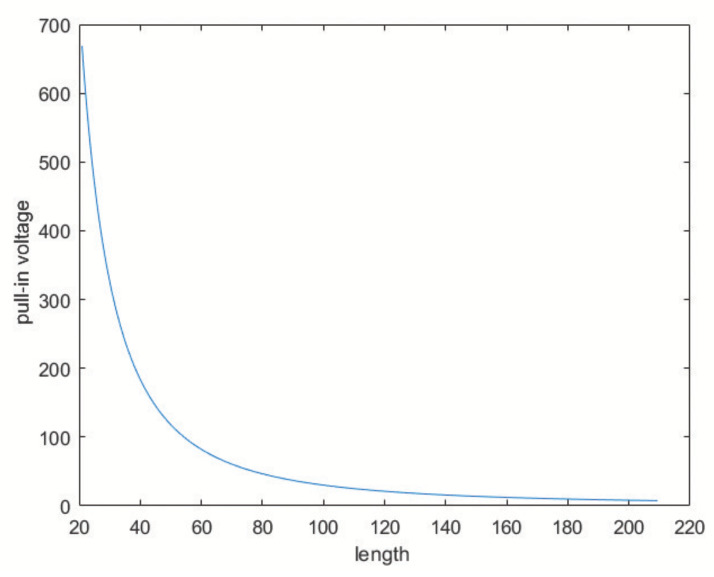
Pull-in voltage versus length of the comb-finger.

Figure 8 displays the trend of the pull-in voltage versus the length of the comb-finger.

#### 4.2.4. Sensitivity Curves

Two different cases can be distinguished when it comes to sensitivity curves:

1. The angular velocity is along the *z*-axis, which results in an in-plane displacement of the sense-mass.
2. The angular velocity is along the *x*-axis or the *y*-axis, which results in an out-of-plane displacement of the sense-mass.

##### Angular Velocity along the *z*-Axis

When an angular velocity is directed along the *z*-axis, the displacement of the sense masses can be separated into two parts:

- A rotation along the center of the sensor depending on the drive-mode.
- A rotation along the connection point with the drive mass depending on the Coriolis force.

The force acting on the center of mass of the sense-mass due to the Coriolis acceleration can be estimated. Indeed, Coriolis acceleration is given by (Equation (25)):

(25) $${\vec{a}}_{coriolis}=-2\vec{\Omega }\times \vec{\dot{x}}$$

Consequently, from the second equation of dynamics (Equation (26)):

(26) $$\vec{F_{c}}=m{\vec{a}}_{coriolis}$$

The velocity associated to the drive-mode can be expressed in cylindrical coordinates as (Equation (27)):

(27) $$\vec{\dot{x}}=\sqrt{x^{2}+y^{2}}\left\{\begin{array}{c} 0\\ \theta *\omega_{d}*c\;o\;s\left(\omega_{d}t\right)\\ 0 \end{array}\right\}$$

Hence, the force acting on a single sense-mass is (Equation (28)):

(28) $$\vec{F}=\left\{\begin{array}{c} -2\sqrt{x^{2}+y^{2}}*\theta *\omega_{d}*cos\omega_{d}t*\Omega *ms\\ 0\\ 0 \end{array}\right\}$$

The displacement is given by (Equation (29)):

(29) $$\vec{x}=\frac{\vec{F}}{\left(\left(j\zeta \left(\Omega -\omega_{s}\right)/ms\right)-\left(\Omega -\omega_{s}\right)2+\omega_{s}^{2}\right)}$$

**Figure 9:**
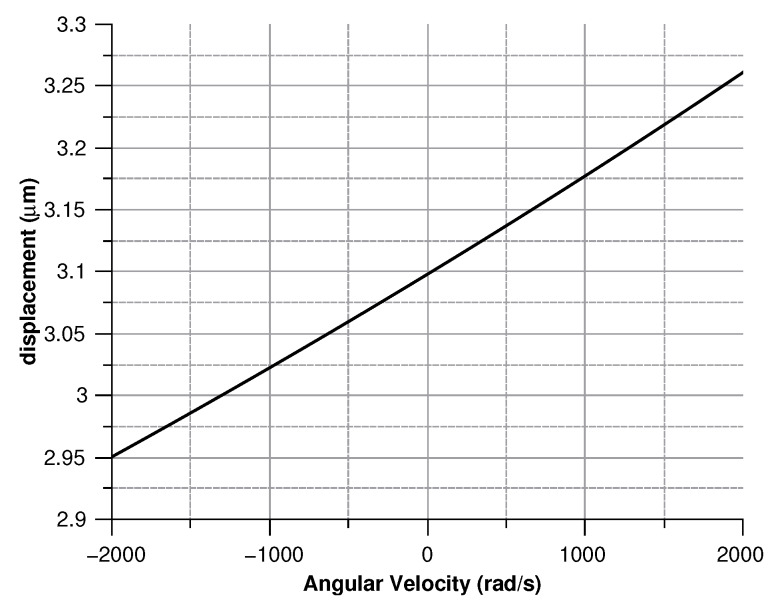
Sense-mass radial displacement vs. angular velocity parallel to the *z*-axis.

**Figure 10:**
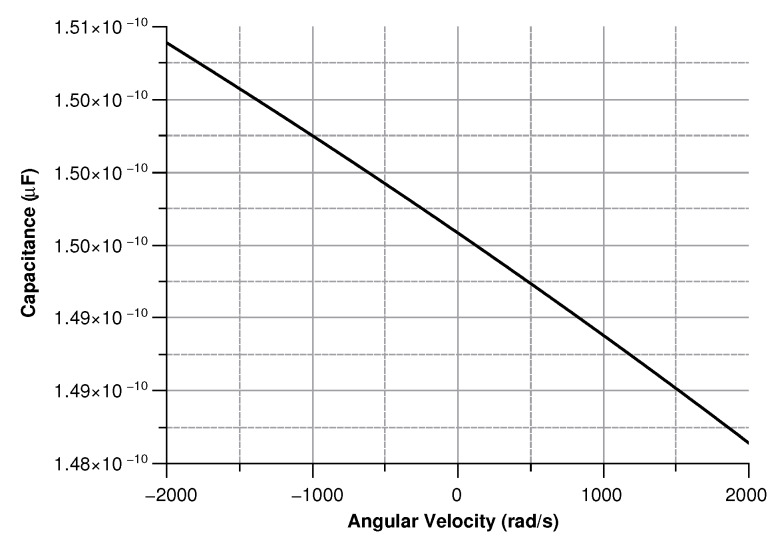
Capacitance between the sense-mass and the ground vs. angular velocity parallel to *z*-axis.

**Figure 11:**
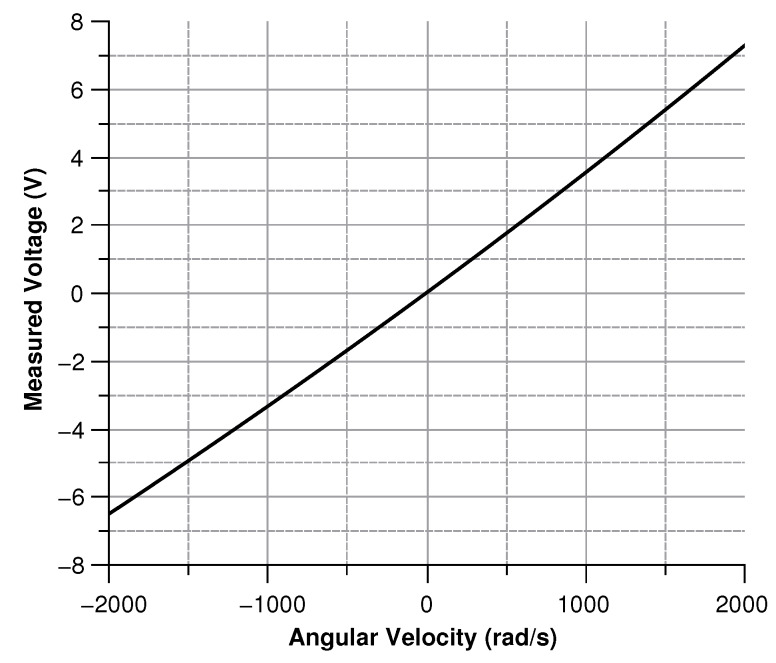
Voltage (according the simulation results) between the sensor and the ground with angular velocity parallel to the *z*-axis.

**Figure 12:**
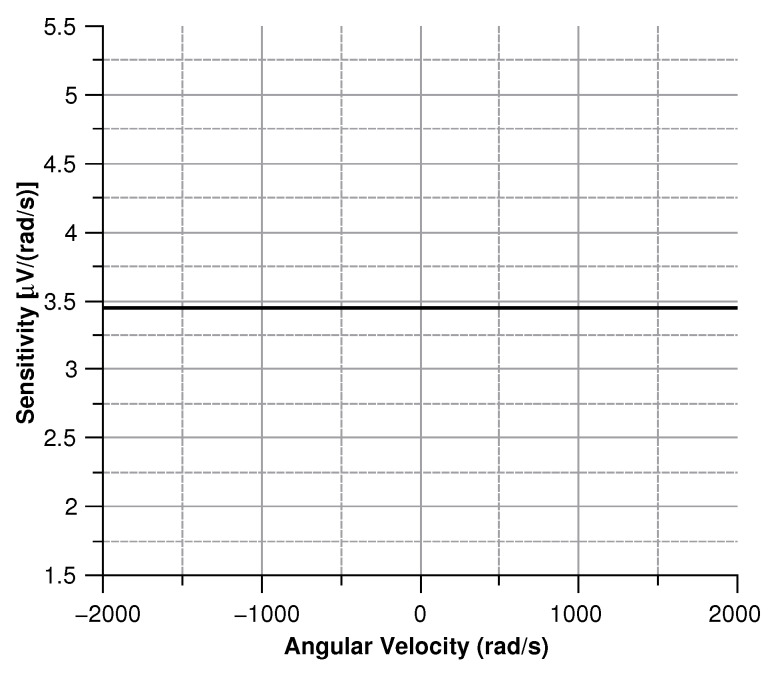
Sensitivity of the sensor vs. the angular velocity parallel to the *z*-axis.

Consequently, the capacitance (Equation (30)) and the modulus of the measured voltage (Equation (31)) are obtained:

(30) $$C=N\varepsilon \frac{t(x_{0}+x)}{g}$$

(31) $$V_{out}=\left(\frac{C(d)}{C_{0}+C\left(d\right)}-\frac{1}{2}\right)V_{in}$$

where in (Equation (30)) the comb-finger were approximated as parallel plate capacitors, *t* is the thickness, *x* the displacement, and *g* the gap between the comb-drivers. In (Equation (31)) $C_{0}$ is the capacitance at zero angular velocity. The results are shown in Figure 9, Figure 10, Figure 11 and Figure 12.

##### Angular Velocity along the *x* and the *y* Axes

When the angular velocity is in-plane, the displacement of the sense masses is out-of-plane. By taking advantage of the same reasoning used for the angular velocity along the *z*-axis, the force (Equation (32)) acting on the sense-mass can be calculated as:

(32) $$\vec{F}=\left\{\begin{array}{c} 0\\ 0\\ -2\sqrt{x_{g}^{2}+y_{g}^{2}}\theta cos\left(\omega_{d}t\right)\Omega \left(cos\left(atan\left(\frac{y_{g}}{x_{g}}\right)\right)-sin\left(atan\left(\frac{y_{g}}{x_{g}}\right)\right)\right) \end{array}\right\}$$

where $x_{g}$ and $y_{g}$ are the coordinates of center of mass of the sense-mass. From the force the displacement can be computed as (Equation (33)):

(33) $$\vec{d}=\frac{\vec{F}}{\left(\left(j\zeta \left(\Omega -\omega_{s}\right)/ms\right)-\left(\Omega -\omega_{s}\right)2+\omega_{s}^{2}\right)}$$

**Figure 13:**
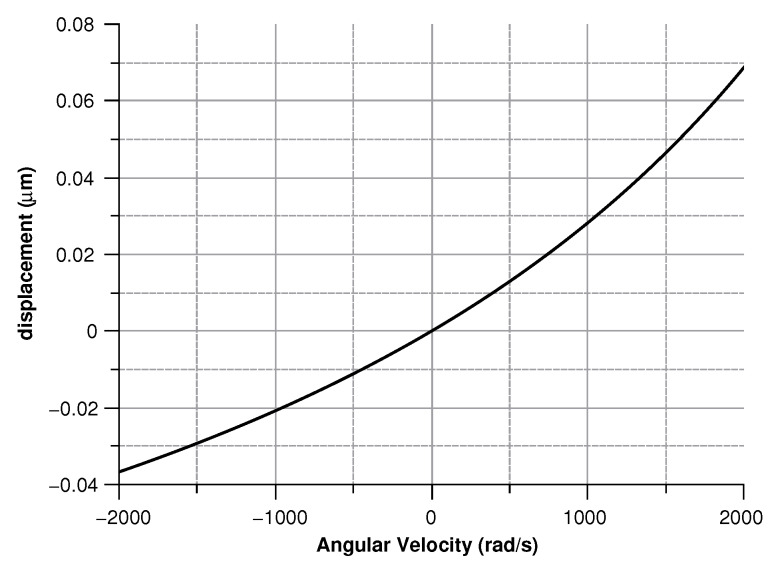
Displacement of the sense mass vs the angular velocity parallel to the *x*-axis.

**Figure 14:**
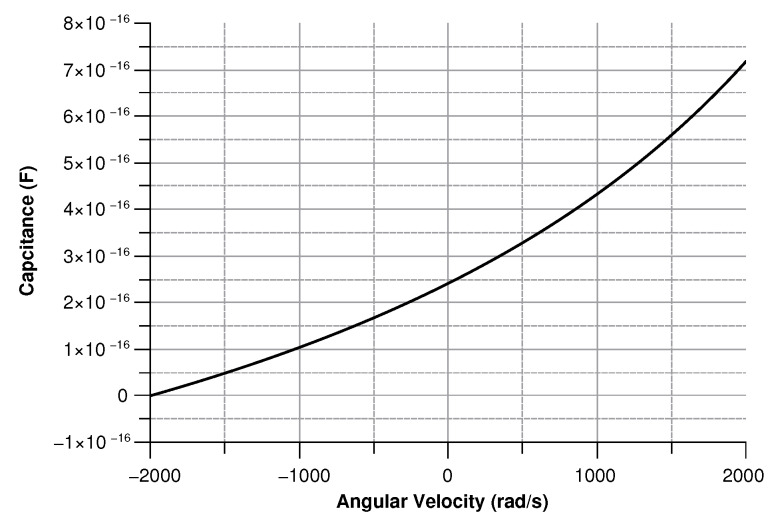
Capacitance between the sense mass and the ground vs the angular velocity parallel to the *x*-axis.

**Figure 15:**
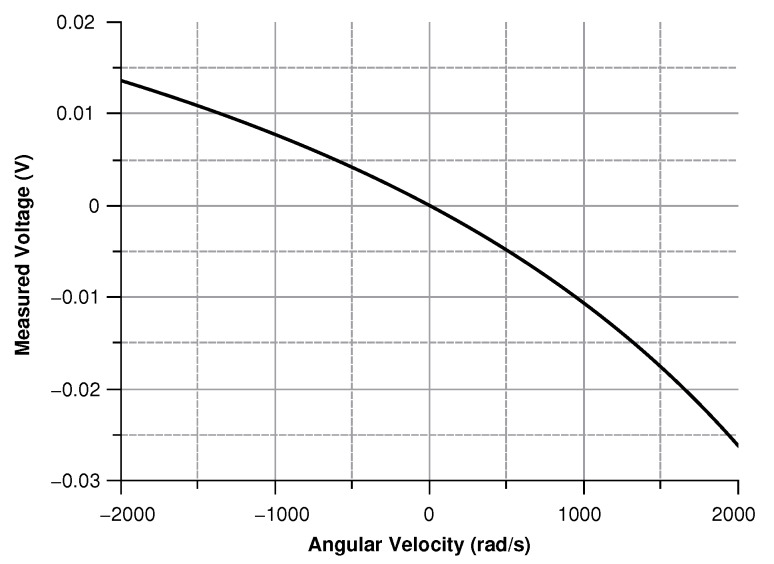
Voltage (according the simulation results) between the sensor and the ground vs the angular velocity parallel to the *x*-axis.

**Figure 16:**
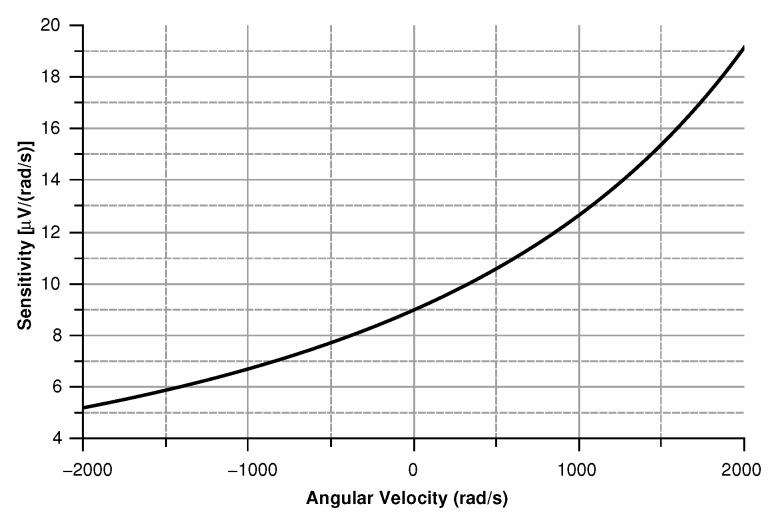
Sensitivity of the sensor with the angular velocity parallel to the *x*-axis.

Figure 13, Figure 14, Figure 15 and Figure 16 illustrate the displacement and the capacitance of a single mass for the case of an angular velocity along the *x*-axis.

#### 4.2.5. Structural Control

The structural calculations were carried out only for the mechanically stressed sections (Figure 2).

##### Section D

**Figure 17:**
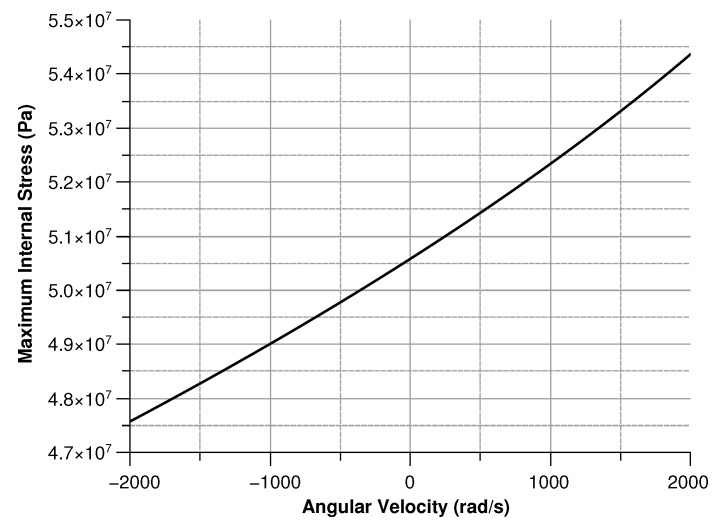
Maximum stress for section D.

Section D is the part of the device where the curved beams and the drive-masses join. For a curved beam, the connection between the beam itself and the frame corresponds to the point of maximum stress. The stress is given by (Equation (34)) [31]:

(34) $$\vartheta_{max}=\frac{3E_{o}}{2l^{2}}x$$

where x is the mass displacement. The maximum stress was computed regardless of the orientation of the angular velocity (along the *x* or *z* axis). The two values were then added up (see Figure 17).

The maximum stress turned out to be 65 MPa. As the maximum allowable stress of polysilicon 1.21 GPa, the safety factor is above 18.

##### Section E

Section E is the final part of the cantilever beam. As Section D, Section E is subjected to a bending moment and the same equation can be used. Spring torsional displacement was neglected in the calculations. The displacement of the tip is 1.3 μm and the maximum stress was found to be

$$\sigma_{max}\approx 233\;\mathrm{Mpa}$$

The safety factor is above 6.

##### Section F

The equivalent stress was calculated by means of the Von Mises’ formula (Equation (35)):

(35) $$\sigma_{eq}=\sqrt{\sigma^{2}+4\tau^{2}}$$

Section F is the ending part of the torsional spring and is subjected to the torque due to the actuation; therefore, the resulting stress is (Equation (36)):

(36) $$\tau_{max}=\frac{T}{W_{t}}$$

where $W_{t}$ is the torsional resistance modulus and, for a circular section, can be calculated through (Equation (37)):

(37) $$W_{t}=\frac{\pi D^{4}}{32}\approx 2.6\;\times 10^{-19}\;m^{4}$$

The maximum tangential stress is thus

$$\tau_{max}\approx 94\;\mathrm{MPa}$$

The equivalent stress is

$$\sigma_{eq}\approx 163\;\mathrm{MPa}$$

Finally, the safety factor is above 9.

## 5. FEM Analysis

The finite method analysis is a numerical technique to reach an approximate result to problems involving partial differential equations with boundary values. The procedure leads to an approximate solution over the defined domain. Then, the domain is divided into smaller and simpler parts, called finite elements. The equations of the finite elements are then assembled into a larger system of equations modeling the entire problem.

In the present study, the FEM analysis was performed through the Comsol software package. In order to increase the accuracy, the etching holes (i.e., the bores on the drive-masses) were also considered in the simulation stage. The solid domain was modeled as a homogeneous and isotropic material. Such a model agrees with the mechanical behavior of polysilicon. The salient parameters of the model are listed in Table 3:

The polysilicon used in the PolyMUMPs^®^ process may be pre-stressed. Thus, the effect of pre-stressing on the mechanical behavior of the device needs to be taken into account. The maximum pre-stress for poly1 is 20 MPa; since the material is considered isotropic the stress tensor describing the pre-stress behavior is (Equation (38)):

(38) $$T=\left[\begin{array}{ccc} 20\;\mathrm{MPa} & 0 & 0\\ 0 & 20\;\mathrm{MPa} & 0\\ 0 & 0 & 20\;\mathrm{MPa} \end{array}\right]$$

**Figure 18:**
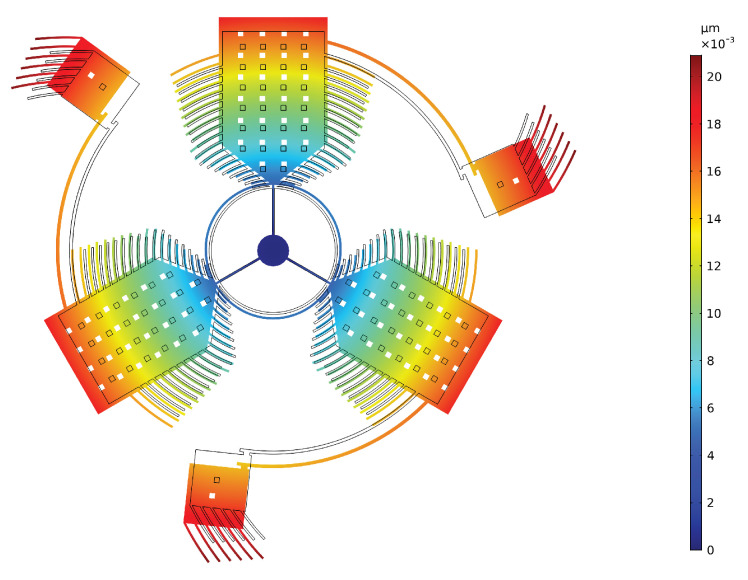
Deformation due to the pre-stress with a scale 1:1000.

The deformation due to the pre-stress is reported in Figure 18.

**Figure 19:**
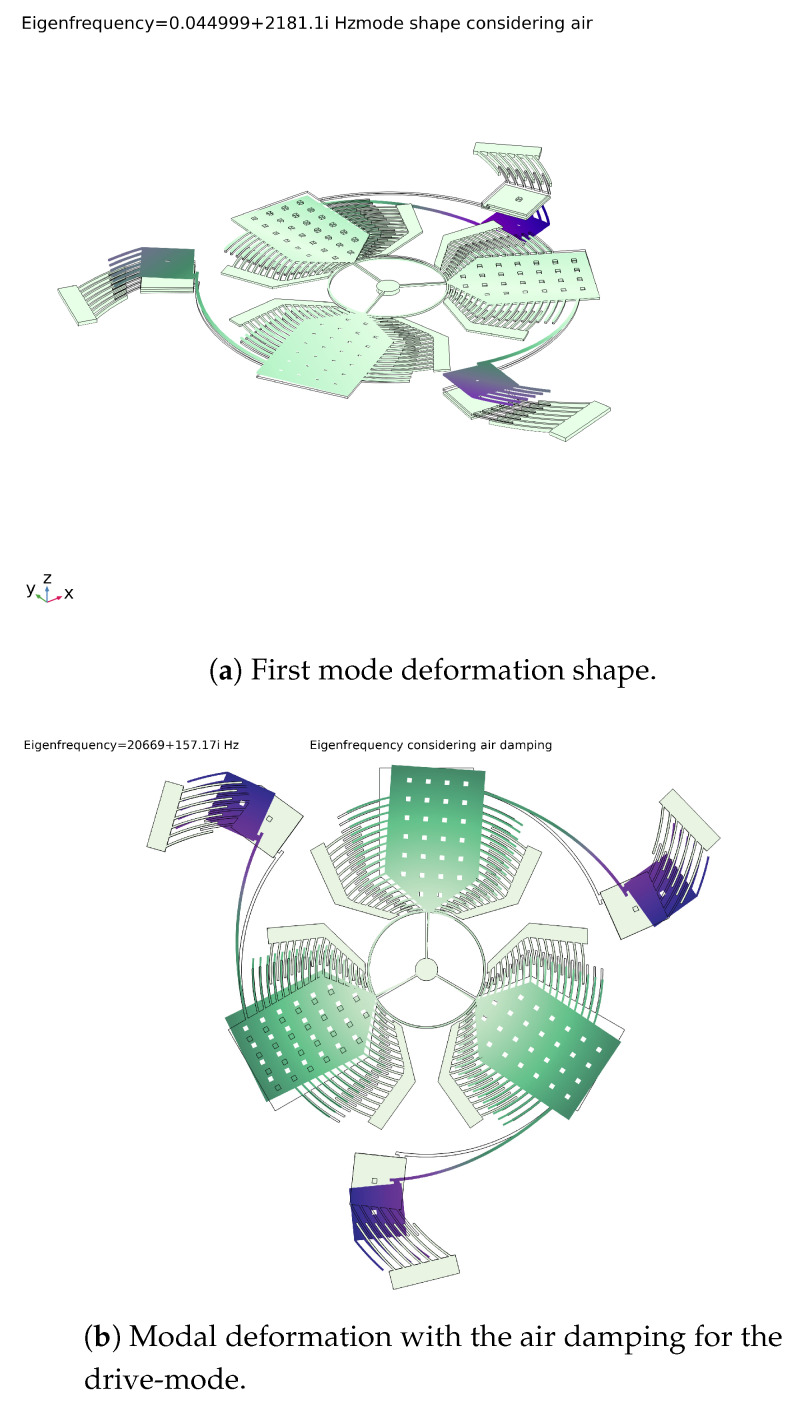
Modal displacement of the 2 working modes: the first regards the out of plane displacement (*x* and *y* sensing) and the second belongs to the in plane sensing (*z* sensing) and the drive-mode considering the effect of the air on the modal displacement.

**Figure 20:**
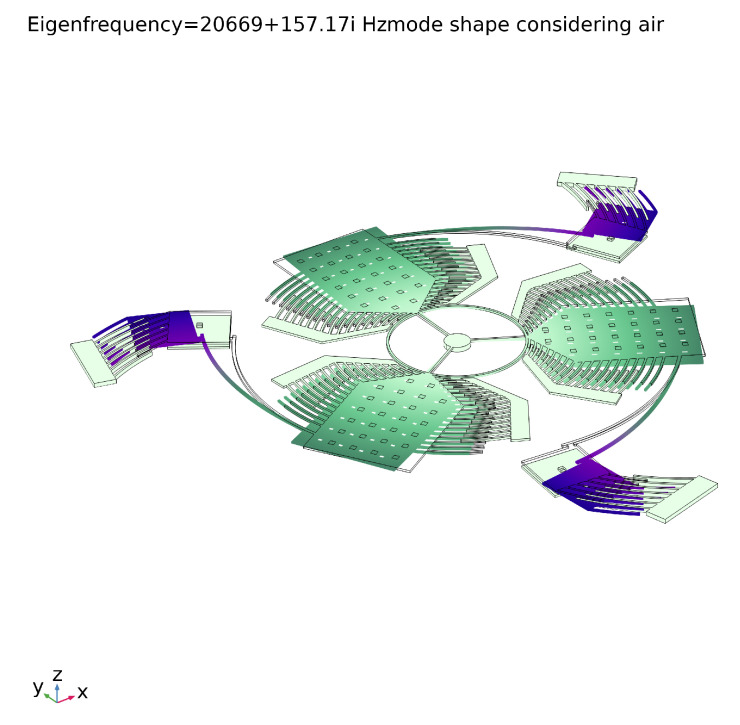
Seventh mode deformation shape.

The modal analysis shows that the only working modes are the first mode, shown in Figure 19a, and the seventh mode of which modal deformation is reported in Figure 20. In particular, the first mode includes the sensing along the x and the y directions allowing the sense-masses to move out of the plane, and the seventh mode includes the sensing along the z direction where the sense-masses present only in-plane displacements.

The maximum percentage deformation on the tip of the sense-mass was found to be about 7×10${}^{-5}\%$. Since the deformation caused by the pre-stress was considerably smaller than the deformation caused by the Coriolis force, the pre-stressing effect was neglected in the next steps of the analysis. The resonance frequency of the structure was calculated for evaluating the deviations from the intended one and the contribution of the sense-masses. Because of the small size of the sensor, the effect of the air mass was also included in the calculation of the resonant frequency. Furthermore, the simulation allowed for the examination of the matching modes at the desired frequency.

**Figure 21:**
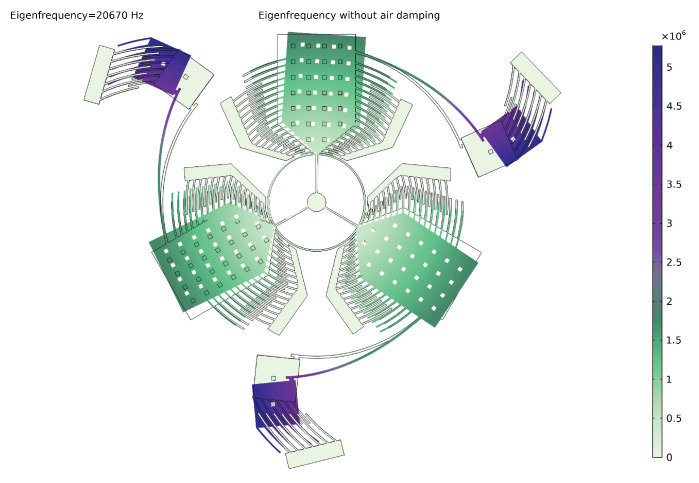
Modal displacement under vacuum for the drive-mode.

Figure 19b and Figure 21 display the modal displacement for the air and no-air cases, respectively. The complex part of the natural frequency describes the damping due to the air. The Q-factor and the time constant of the sensor could also be calculated (Equation (39)):

$$Q=\frac{|\omega_{n}\;|}{\frac{(img\left(\omega_{n}\right)}{2})}\approx 66$$

(39) $$\tau =\frac{2Q}{|\omega_{n}|}\approx 0.0064\;s$$

**Figure 22:**
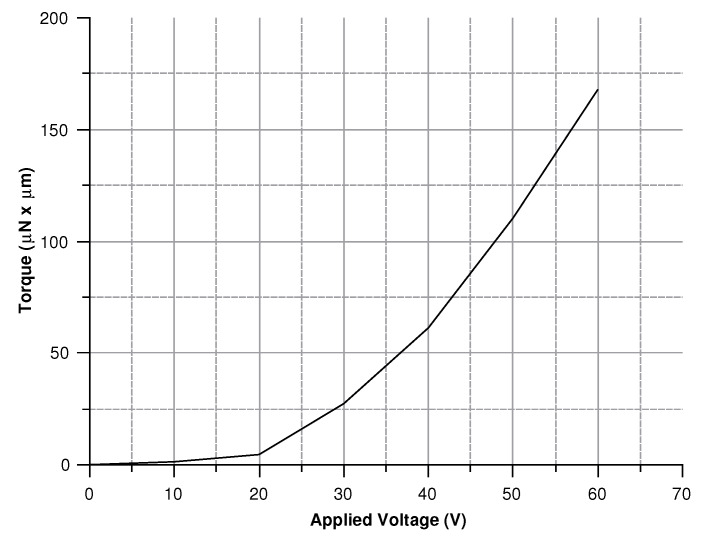
Torque for voltages in 0–60 V.

**Figure 23:**
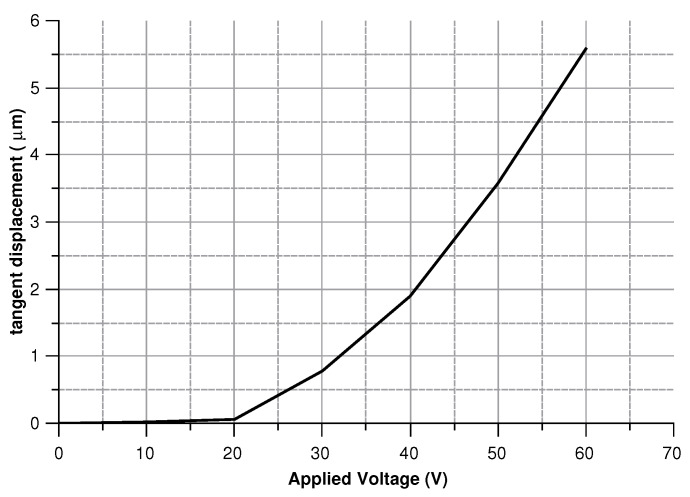
Circumferential displacement of the structure for voltages in 0–60 V.

**Figure 24:**
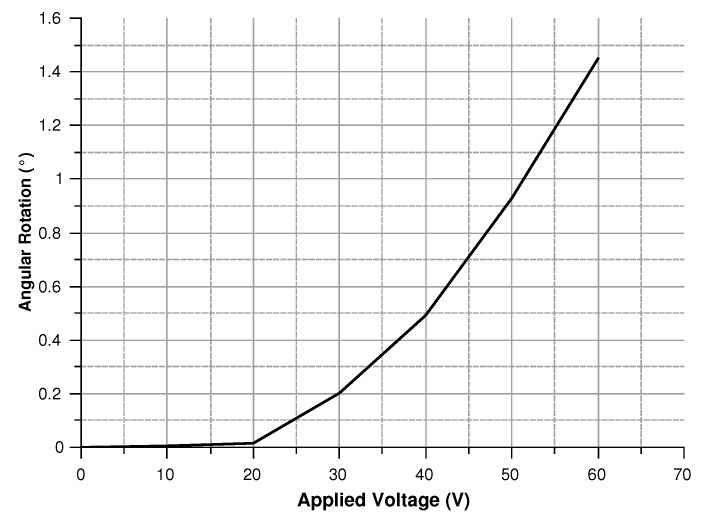
Angular rotation of the structure for voltages in 0–60 V.

Modeling the electrostatic force acting on the structure corresponds to simulate the actuation process. Hence, a voltage between 0 and 60 V was set to compute the torque acting on the structure, the angular rotation, and the circumferential displacement. Results are presented in Figure 22, Figure 23 and Figure 24.

### 5.1. Complete Behavior of the Sensor

A −2000–2000 rad/s angular velocity range was employed in the simulation, which is standard for these calculations. The nomenclature used in the next sections follows the one illustrated in Figure 2.

#### 5.1.1. Angular Velocity along the *z*-Axis

**Figure 25:**
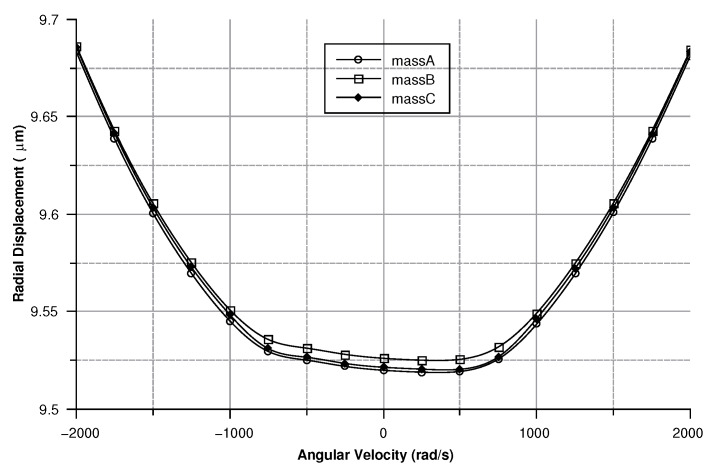
Modulus of the radial displacement for the sense-mass tip.

When the angular velocity is directed along the *z*-axis, the displacement of the sense masses points along the connection with the drive-masses. Figure 25 shows the displacement modulus of the sense-mass tip.

For a clockwise angular velocity (i.e., negative in the graph) the displacement of the sense-mass is in phase with respect to the movement of the drive-mass. On the other hand, for a counter-clockwise (positive in the graph), the displacement of the sense-mass is in counterphase with respect to the movement of the drive-mass. The displacement resulted to be 10 μm off compared to the predicted value.

**Figure 26:**
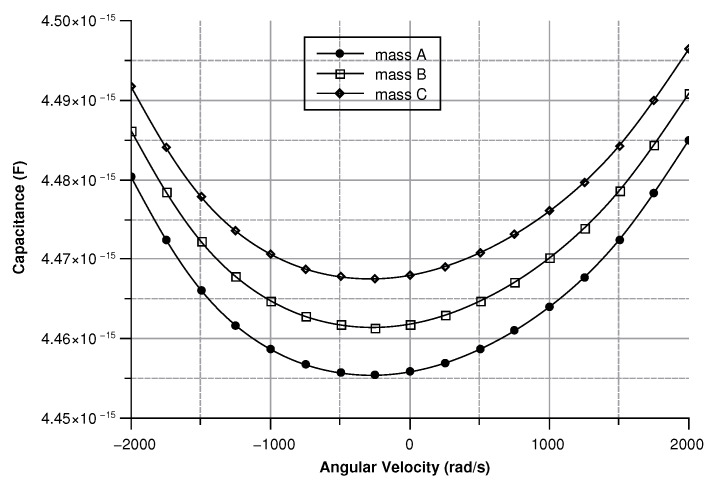
Capacitance due to the displacement of the sense-mass tip.

Coupling between the drive-mode and the sense mode due to matching modes accounts for this effect, as can be seen in Figure 21. Nonetheless, the quadrature error is constant and it can be easily removed. Figure 26 depicts the capacitance variation between the sense-mass and the ground.

As expected, the capacitance changes linearly as the displacement changes. The difference in the starting points of the three masses is brought about by the the slight asymmetry of the sensor. Nevertheless, this issue can be eliminated by setting three different values of the capacitance on each of the Wheastone-bridge used to measure the capacitance variation. The signal coming from the capacitive Wheastone-bridge can be easy calculated as shown by equation (Equation (40)):

(40) $$V_{out}=\frac{C(d)}{C_{0}+C\left(d\right)-1/2)}V_{in}$$

where $V_{out}$ is the measured voltage, $V_{in}$ is the supplied voltage, $C_{0}$ is the capacitance of the three capacitors of the Wheastone-bridge, and *C*(d) is the capacitance due to the sense-mass.

**Figure 27:**
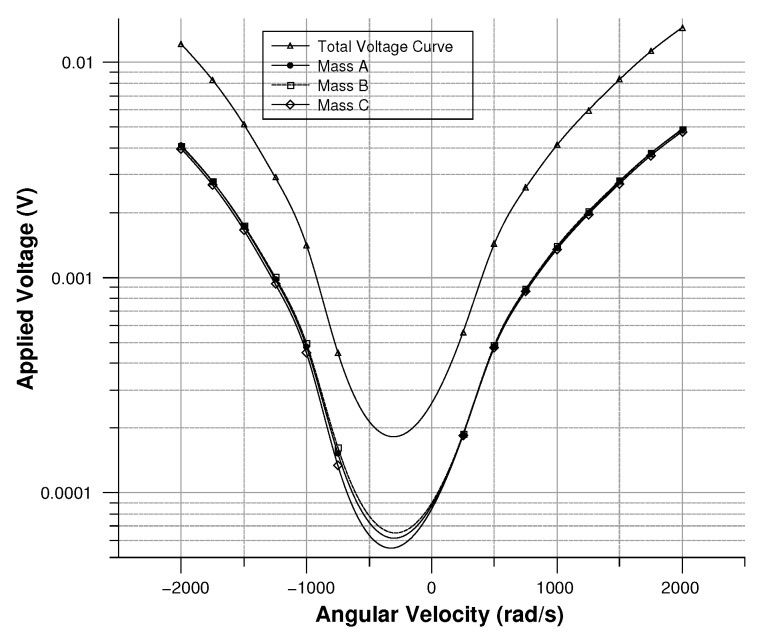
Modulus of the measured voltage for each of the three-sense mass.

**Figure 28:**
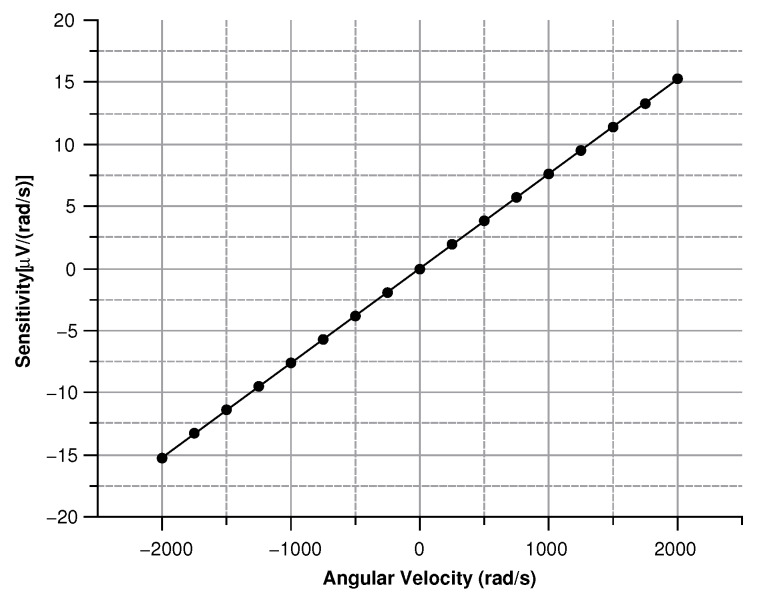
Sensitivity curve for sensing along the *z*-axis.

As shown in Figure 27 modulus of the measured voltage depends quadratically on the angular velocity. The sensitivity curve can be obtained by deriving the curve in Figure 27 with respect to the angular velocity (Figure 28).

**Figure 29:**
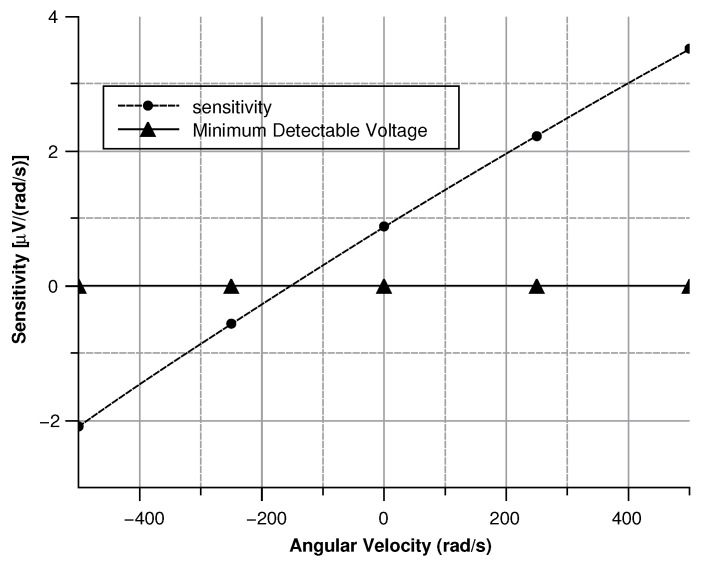
Enlargement of the sensitivity curve around the minimum point.

The minimum in the sensitivity (Figure 29) curve corresponds to 0.8 μV/(rad/s), a value well above the lower detection limit of most voltage measuring instruments.

#### 5.1.2. Angular Velocity Along the *x* and *y* Axis

**Figure 30:**
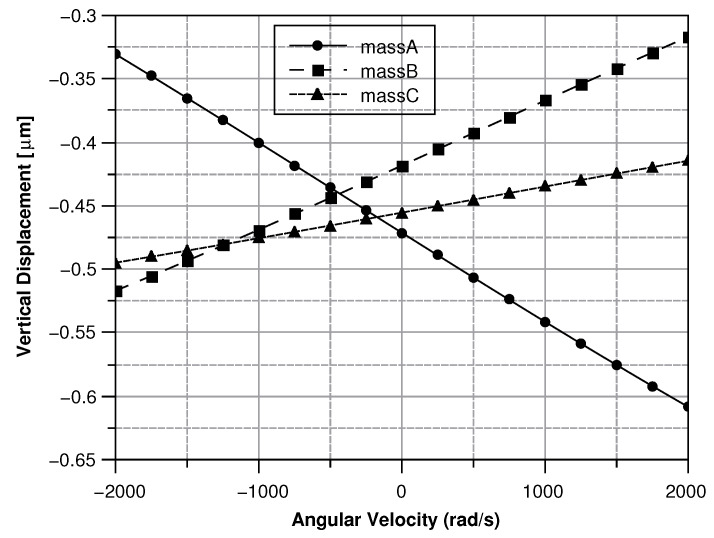
Vertical displacement of the three sense-masses for an angular velocity along the *x* axis.

**Figure 31:**
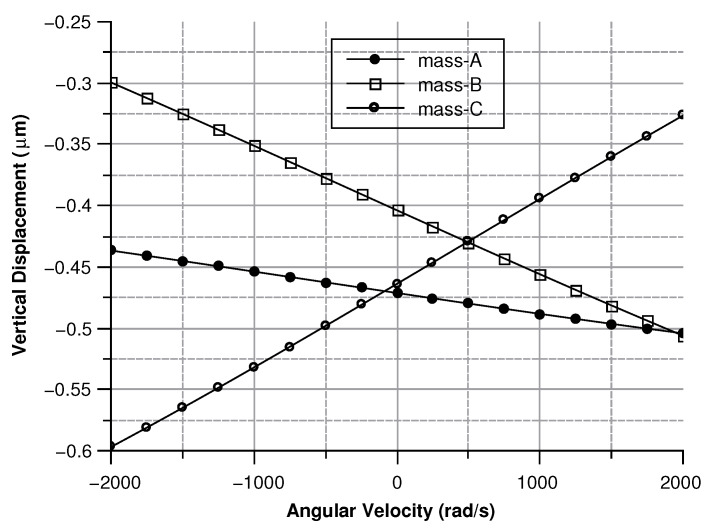
Vertical displacement of the three sense-masses for an angular velocity along the *y* axis.

Because of their great similarity the *X* or the *Y* axis cases are described together. In both cases (see Figure 30 and Figure 31) As previously mentioned, this effect reflects into the output signal and can be used to determine the direction of the angular velocity. For instance, if the angular velocity is directed along the x-axis the mass B exhibits the smallest displacement. Conversely, if the angular velocity is directed along the y-axis mass A shows the lowest displacement.

**Figure 32:**
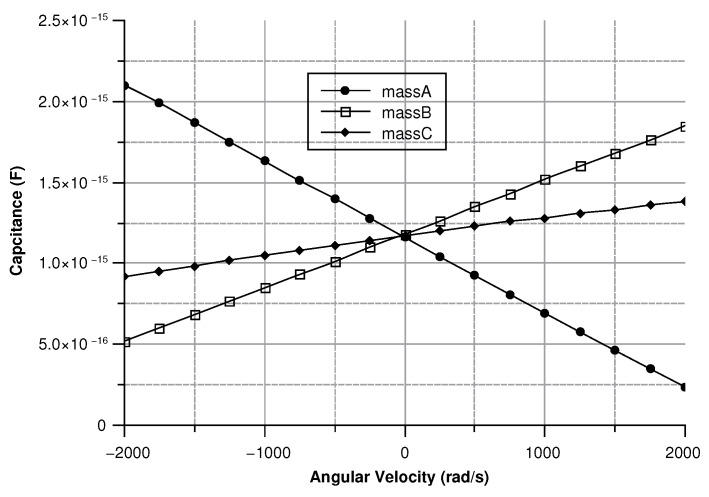
Capacitance of the three sense masses with an angular velocity along the *x* axis.

**Figure 33:**
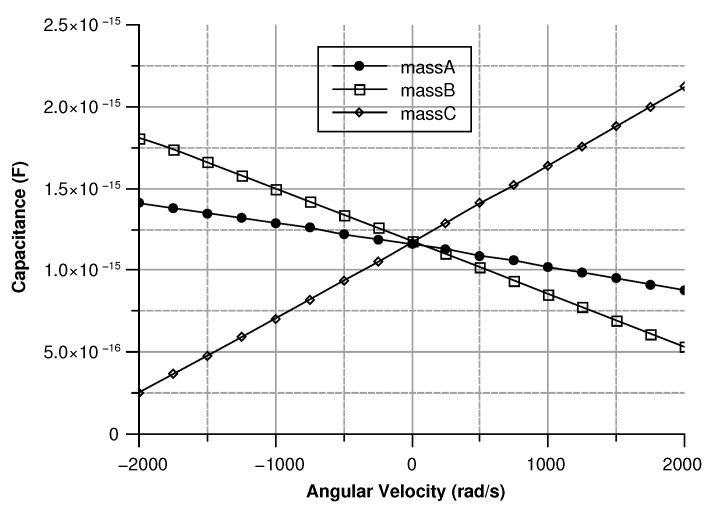
Capacitance of the three sense masses with an angular velocity along the *y* axis.

**Figure 34:**
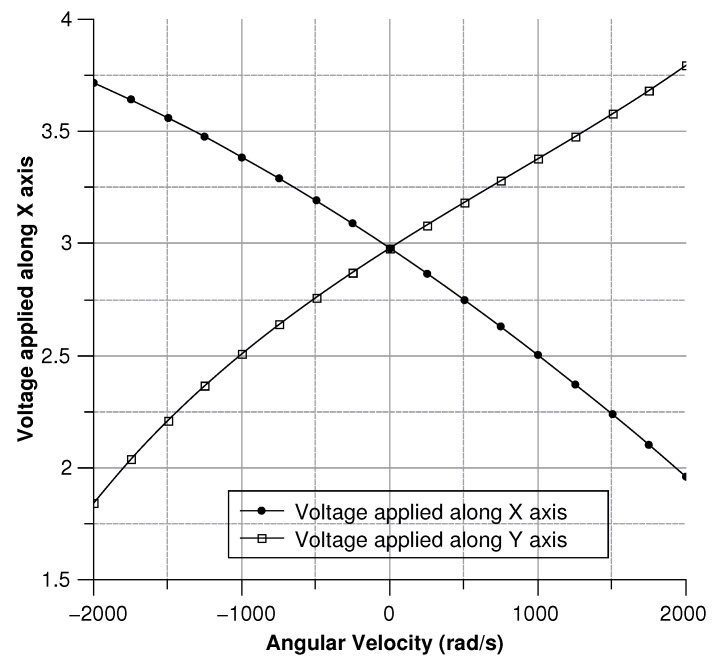
Voltage variation for an angular velocity along the *x*- and *y*-axis.

Since the three sense-masses can be modeled as three plane capacitors, the capacitance (Figure 32 and Figure 33) follows along the trends of the displacement. As expected, the capacitance behaves linearly with respect to the displacement. The modulus of the output voltage was computed through (Equation (41)):

(41) $$V_{out}=\frac{\left(C_{A}*C_{B}-C_{c}*C_{0}\right)}{\left(\left(C_{A}+C_{B}\;\right)*\left(C_{c}+C_{0}\;\right)\right)}$$

the results are shown in Figure 34.

**Figure 35:**
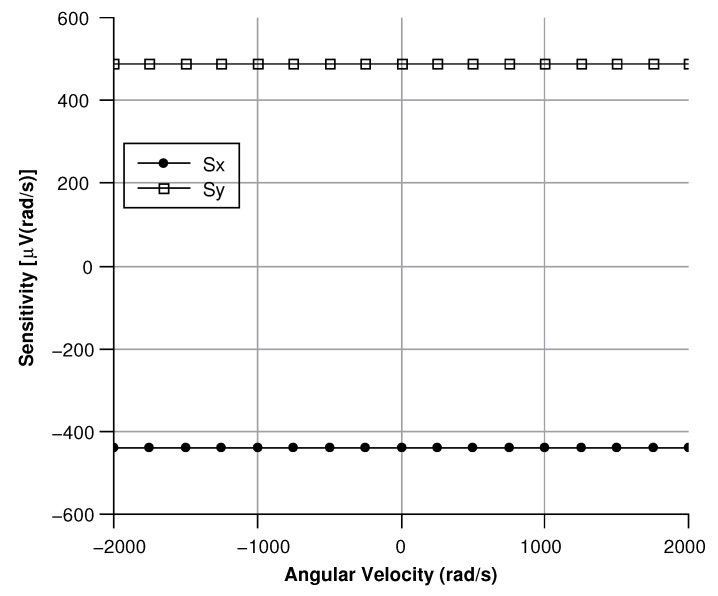
Sensitivity curve for angular velocities directed along the *x*- and *y*-axis.

By differentiating the graphs in Figure 34 with respect to the angular velocity, the sensitivity curves can be obtained (see Figure 35).

## 6. Comparison with Similar Devices

Commercial and academic tri-axis gyroscopes are usually attained assembling together three orthogonal single-axis gyroscopes. A monolithic gyroscope reduces the chance of alignment error during the measurements. Studies have been devoted, both in industry and academia, to devise and actually produce monolithic tri-axial gyroscopes.

The characteristics of two gyroscope (one commercial, one devised by a university research group) were compared and contrasted to the device introduced in this paper (see Table 4 and further discussion).

Although the sensor presented in this article shows low performances compared to the other two, it can take advantage of the small size. Indeed, reduced dimensions bring about a simpler design, an increase in the stability of the sensor, and less chance of construction errors. In addition, the low weight and the greater range of measurable angular velocities make the present sensor especially well-suited for applications where extremely fast angular velocity changes are not unusual such as automotive and aero-space.

Future work will focus on the production of the sensor through industry-standardized surface micromachining. Needless to say, such an approach leads to a considerable cost reduction of MEMS for real IC monolithic integration.

## 7. Conclusions

A tri-axial gyroscope was designed for angular velocity measurement purposes. Its silicon-based structure is comprised of suspended masses anchored to the substrate by means of cantilever beams. The design was devised to guarantee the same resonant frequency for both actuation and sensing operations. This feature was introduced in order to amplify the signal stemming from the imposed force. The present project aimed at proposing an electro-mechanical system capable of implementing both complex movements and detection of small forces. The main innovation proposed by the present gyroscope is the immense size shrinking compared to commercially available counterparts. Sensor planarity is also an important characteristic included in the design of this gyroscope. Indeed, the possibility of fabricating the device by two polysilicon layers at most may cause an astonishing drop in the production cost.

The design of the sensor was divided in three different sections associated to three different development stages:

1. A first guess solution predicated on basic formulas in order to provide a starting point for the successive steps.
2. An algorithm based on an iterative procedure to refine the first guess solution according to the fabrication process constraints.
3. The validation of the final solution by a FEA (Finite Element Analysis) software.

The final output is a fast responding (to angular velocity changes) sensor with a sensitivity of 0.4 μV/(rad/s) and a mass of 0.47×10${}^{-3}$ g, which implies a very low observational error. The device is especially well-suited for surface micromachining-based fabrication and for application requiring a precise, rapid, and low-cost angular rate measurement such as the automotive and automation industries. 

## Figures and Tables

**Table 1:** Mechanical properties of the sensor (bulk silicon).

Name	Value
Young Modulus	158 GPa
Density	2380 Kg/m${}^{3}$
Fracture strength	1.21 GPa
Poisson’s module	0.22
Resonance frequency	21 KHz

**Table 2:** Geometrical dimensions of the sensor.

Name	Value
Sensor Area	600 μm × 600 μm
Sensor Thickness	2 μm
Drive Mass Length	100 μm
Sense Mass Length	50 μm
Number of actuation comb-drives	15
Number of sense comb-drives	6
Sense cantilever beam length	182 μm
Sense cantilever beam width	3 μm
Drive cantilever beam length	60 μm
Drive cantilever beam width	2 μm
Connector radius	7.5 μm
Annulus connector radius	60 μm

**Table 3:** Polysilicon mechanical characteristics.

Name	Value
Young Modulus	160 GPa
Density	2320 Kg/m${}^{3}$
Poisson’s module	0.23

**Table 4:** Comparison between the three gyroscopes.

	Xia Gyroscope	L3G4200D	Tri-Axis Planar Gyroscope
	(Academic Research)	(Commercial)	(Present Work)
Natural frequency	8 KHz	20 KHz	20 KHz
Q-factor	500	200	60
Minimum angle	$1.82\times 10^{-5}$ (${}^{\circ }$/s)	$57\times 10^{-3}$ (${}^{\circ }$/s)	$2.5\times 10^{-3}$ (${}^{\circ }$/s)
Sensitivity	$1.89\times 10^{-16}$ F/(${}^{\circ }$/s)	70 m (${}^{\circ }$/s)/digit	$7.55\times 10^{-18}$ F/(${}^{\circ }$/s)
Sense linearity	0.17%	0.2%	0.7%
Dimensions [μm]	9200×9200×60	2000×2000×40	600×600×2
Range	±500 (${}^{\circ }$/s)	±2000 (${}^{\circ }$/s)	±115,000 (${}^{\circ }$/s)
